# Infections with *Schistosoma mansoni* and geohelminths among school children dwelling along the shore of the Lake Hawassa, southern Ethiopia

**DOI:** 10.1371/journal.pone.0181547

**Published:** 2017-07-18

**Authors:** Bamlaku Tadege, Techalew Shimelis

**Affiliations:** 1 Hawassa University, College of Medicine and Health Sciences, School of Medicine, Microbiology and Parasitology Unit, Hawassa, Ethiopia; 2 Hawassa University, College of Medicine and Health Sciences, Department of Medical Laboratory Science, Hawassa, Ethiopia; Brighton and Sussex Medical School, UNITED KINGDOM

## Abstract

**Background:**

Infections with *Schistosoma mansoni* and soil-transmitted helminthes (STHs) are major public health problems in Ethiopia. However, information was scarce on the current status of these infections to guide an intervention in the study area. Therefore, this study was conducted to assess the prevalence of infections with *S*. *mansoni* and STHs and associated factors among school children in southern Ethiopia.

**Methods:**

This cross-sectional study investigated children who were attending the Finchawa and Tullo junior elementary schools and were residing along the shore of the lake Hawassa in January and February, 2015. A total of 374 students were selected using systematic random sampling technique. Data on socio-demography and related factors was collected using structured questionnaires. A single stool sample was collected from each child and processed using formol-ether concentration technique and examined microscopically for parasites’ ova/larva.

**Results:**

The prevalence of parasitic infection with one or more than one helminthiasis was found to be 67.9%. Seven different types of helminths were identified and the most prevalent parasites were *Ascaris lumbricoides* (44.4%), followed by *S*. *mansoni* (31%), *Trichuris trichiura* (11%), and hookworms (7.7%). The rate of infection with STHs was 52.4%. Single, double, triple and quadruple infections were 42.2, 22.5, 2.4, and 0.8%, respectively. Children who practiced open-field defecation (AOR, 3.6; 95% CI 1.6–8.0; p = 0.001) and had not always washed their hands before eating a meal (AOR, 5.0; 95%CI 2.15–11.7; p <0.001) were more infected with STHs. Moreover, the rate of *S*. *mansoni* infection was significantly higher among children who were attending the Finchawa school (AOR, 2.13; 95% CI 1.31–3.46; p = 0.002), aged 11–15 years (AOR, 1.97; 95% CI 1.22–3.19; p = 0.006), had swum in the lake Hawassa (AOR, 2.73; 95%CI 1.20–6.17; p = 0.016), and had involved in irrigation-related activities (AOR, 1.68; 95%CI 1.04–2.71; p = 0.034).

**Conclusion:**

The study showed high prevalence of STHs and moderate rate of schisotosomiasis. Mass deworming twice a year for STHs and once every two years for *S*. *mansoni*, further to strengthening other prevention measures, is critically needed to reduce these infections to inconsequential level.

## Introduction

Intestinal parasitic infections are the major medical and public health problems in many parts of the world [1]. Schistosomiasis and soil-transmitted helminthiasis are among the most widely distributed neglected tropical diseases (NTDs) that affect people living in communities with poor access to safe water and sanitary and health facilities [2–4]. It was estimated that more than a billion people globally had chronic infections of geohelminths/soil-transmitted helminths (STHs) [5]. The global burden of infections with *Ascaris lumbricoides*, hookworms, and *Trichuris trichiura* are about 1000, 900–1,300, and 500 million, respectively [6–8]. Schistosomiasis is the most deadly NTD and ranks second next to malaria as a cause of mortality. An estimated 700 million people in 76 countries are at risk of schistosomiasis, and 240 million people are already infected. About 85% of the infections occur in Africa where a yearly estimated death is 280,000 people [9].

In addition to the morbidity and mortality associated with intestinal helminthiasis, infections have the consequences of impairing physical growth and development, mental function, verbal ability, and cognitive domain [10, 11]. It has been estimated that 400 million infected school-age children are often physically and intellectually compromised by anaemia, leading to attention deficits, learning disabilities, school absenteeism and higher dropout rates [12].

Most infections with intestinal parasites occur in poor communities where the biophysical, cultural and environmental factors favor transmission [13]. Low standard of living, poor personal hygiene, unsanitary west management, unsafe and inadequate water supply are some of the factors that allow intestinal parasites and other communicable diseases to flourish in developing countries. A rapid spread of schistosomiasis is associated with water resource development and intensive population movements [9]. Younger children are particularly at higher risk of contracting STHs as they habitually play in faecally contaminated ground and place soiled fingers into their mouth [14]. Moreover, their active involvement in activities that increase an exposure to water bodies put children at increased risk of getting schistosomiasis [15].

Ethiopia is one of the most affected countries with helminths where estimates of infections with *A*. *lumbricoides*, *T*. *trichiura*, hookworms and *S*. *mansoni* were 26, 21, 11 and 5 million, respectively [13]. The altitudinal category for optimal transmission of *S*. *mansoni* and geohelminths is between 1000 and 2000 meters, and most endemic localities in the country are located in this altitudinal range [16]. The national prevalence of ascariasis, trichuriasis and hookworm infection was estimated to be 37, 30 and 16%, respectively [17]. Moreover, in a nationwide survey (1988–89), the prevalence of schistosomiasis mansoni was shown to be 25% [18]. Studies that investigated school children in different localities of the country reported prevalence ranging from 5.7 to 83.4% for *A*. *lumbricoides*, 1.7 to 86.4% for *T*. *trichiura*, 2.2 to 60.2% for hookworms [19–25], and 21.2 to 89.9% for *S*. *mansoni* [24, 26–28].

In the effort to reduce the clinical and epidemiological impacts of STHs in Ethiopia, the Federal Ministry of Health in collaboration with partners has been administering mass chemotherapy targeting under-five children since 2010 as a component of the Enhanced Outreach Strategy (EOS) [13]. Moreover, through the health extension program, interventions are on progress such as proper and safe excreta disposal system, safe solid and liquid waste management, water supply safety measures, food hygiene and safety measures, healthy home environment and personal hygiene. Communities in the study area have been involved to support the implementations of these interventions. The ministry has also launched a nationwide mass deworming program against schistosomiasis and STHs, which targets school children; but the current study was carried out just before the start of this program in the study area. However, information was scarce on the current status of the infections to help make comparisons and guide intervention efforts. This study aimed to assess the prevalence of *S*. *mansoni* and geo-helminths and associated risk factors among school children dwelling along the shore of the Lake Hawassa in southern Ethiopia.

## Materials and methods

### Study setting and design

A cross-sectional quantitative type of study was conducted at Finchawa and Tullo junior elementary schools from January to February, 2015. The respective schools are located in Finchawa and Tullo *kebeles* (administrative units) in Tulla District of the Southern Nation, Nationalities and Peoples’ Region (SNNPR). These are rural *kebeles* located along the shore of the Lake Hawassa and have a population size of 14138 and 9815, respectively. Selection of the schools was based on the presence of water contact points along the shore of the lake. The altitude of the area is 1697 meter above sea level and has a mean annual temperature 20.9°C and mean rainfall 997.6 mm [29]. Social service institutions in the area include, 1 non-governmental major health center, 1 health post and 5 schools (1 kindergarten, 3 junior elementary and 1 secondary). The health center provides clinical, diagnostic and treatment services for its catchment population. With the support of the health extension program, most households owned locally constructed pit latrines. Hand pump water supplies were available at the *Kebeles* although residents also utilize the lake water for various domestic, laundry, irrigational and recreational purposes. There are adequate numbers of latrines and pipe water supplies to be utilized by the students at the schools. However, it was observed during the data collection period that latrines in the schools were improperly utilized as the sitting site and surroundings were highly polluted with feaces. Moreover, we observed in the schools that most students did not wash their hands after using latrines. No latrine was available nearby the lake; yet a number of children and adults played and practiced fishing, irrigation, swimming and washing clothes using the lake water. Fecal material was seen around the lake water, which raises concern regarding the sanitation of the lake.

### Population

The total number of students attending Finchawa and Tullo junior elementary schools were 1665 and 1163, respectively. Thus, the source population size would be 2828 students attending the two schools during the study period. However, students who had stayed in the *kebeles* for less than a year period or had taken anti-helminthic drugs within a month prior to the time of data collection were excluded.

### Sample size and technique

Sample size was estimated using a single population proportion formula with EPI-Info version 7. Considering the source population size 2828, prevalence of trichuriasis 46% [23], level of confidence 95%, margin of error 5%, and response rate 90%, sample size was calculated to be 374. We used prevalence of trichuriasis for the calculation as it generates maximum sample size compared to rates reported for other helminthes in the study area. The students were first stratified according to their school and educational level (Grade 1 to 8). Sample size was proportionally allocated for each class and grade, taking the total number of students in each category in to consideration. Class rosters were used as a sampling frame and a systematic sampling technique was employed to select study subjects.

### Data collection

A two-day training was given for four diploma nurses, who spoke the local language (Sidamigna). Training focus on explaining the purpose of the study, obtaining consent from parents/guardians and how to interview them using questionnaire. Data collectors visited students’ home and interviewed parents/guardians on socio-demography and risk factors of intestinal parasites using structured questionnaires. The principal investigator checked the completeness of gathered data and took correction in instances missed information was detected. Moreover, a single stool sample was collected from each study participant and preserved in 10% formalin solution. Then, specimens were transported to the laboratory of Hawassa University Referral Hospital and processed using formol-ether concentration technique. Two slides were prepared from each fecal deposit and read microscopically for eggs/larva by two independent laboratory technologists (Bachelor of Science Degree holders) with at least two years’ work experience. In instances when results between the two laboratory personnel were discordant, the principal investigator examined those samples and results would be conclusive.

### Data analysis

Data was entered and analyzed using SPSS version-16 software. Results were summarized using frequencies and proportions, and presented with tables. Crude odds ratio (COR) from binary logistic regression analysis help assess the association between independent and outcome variables. Adjusted odds ratio (AOR) was also computed using multivariable logistic regression analysis taking all factors yielding a p-value ≤ 0.25 in bivariate analysis. A p-value ≤ 0.05 was considered to be statistically significant.

### Ethical clearance

The study was ethically approved by the Institutional Review Board of Hawassa University College Medicine and Health Sciences. Supportive letter was written to the respective school directors to get permission and select the study participants, to arrange specimen collections schedule and to use institutional facilities. After selecting the study participants, data collectors conduct home- to- home visit to obtain written consent from parents/guardians. Assent was also obtained from students after their parent/guardian consented. Finally, data collectors interviewed parents/guardians using questionnaires. Children who had any intestinal helminthic infection were treated according to the national guideline [30].

## Results

A total of 384 school children were recruited into the study, but a complete data was obtained from 374 students. Ten students were excluded because they were unable to provide a stool sample at the time of data collection. Students from Finchawa Elementary School accounted 51.6% of the study participants compared to 48.4% in Tullo Elementary School. The majority of the participants were boys (62.8%) and in the age range 10–15 years (53.7%) (mean, 11.2 years). Among the study participants, 96.5% had traditional pit latrines at home, yet 37.7% had practiced open- field defecation. The habit of not washing hands after return from latrine and before eating a meal was 50.8 and 43%, respectively. Students who had not periodically trimmed their fingers’ nail and wear a shoe frequently accounted 52.7 and 29.4%, respectively (Table 1).

**Table 1:** The distribution of soil-transmitted helminthiasis by socio-demography and other factors among school children in southern Ethiopia, 2015.

Characteristics	Total %	Number (%) STHs infected	COR (95% CI)	AOR (95% CI)	P value
Sex					
Male	235(62.8)	113(48.1)	0.61(0.39–0.93) **	0.97(0.55–1.7)	0.955
Female	139(37.2)	84(60.4)	1	1	
Age (years)					
5–10	173(46.3)	91(52.6)	0.99(0.66–1.49)	-	
11–15	201(53.7)	106(52.7)	1		
Address					
Tullo	181(48.4)	95(52.6)	1		
Finchawa	193(51.6)	102(52.8)	1.02(0.67–1.52)	-	
Latrine at home					
Yes	361(96.5)	188(52.1)	1	1	
No	13(3.5)	9(69.2)	2.1(0.62–6.84)	2.15(0.44–10.4)	0.340
Defecation site at home					
Latrine	233(62.3)	73(31.3)	1	1	
Open field	141(37.7)	124(87.9)	17.5(9.68–31.5) **	3.82(1.72–8.51) **	0.001
Washing hand after latrine					
Yes	1849(49.2)	44(23.9)	1	1	
No	190(50.8)	1531(80.5)	14.02(8.52–23.1) **	1.86(0.83–4.15)	0.131
Washing hand before meal					
Yes	213(57)	57(26.8)	1	1	
No	161(43)	140(87)	19.76(11.3–34.6) **	5.13(2.19–12.0) **	<0.001
Trimming nail periodically					
Yes	177(47.3)	50(28.2)	1	1	
No	197(52.7)	147(74.6)	7.89(4.97–12.5) **	1.67(0.89–3.14)	0.106
Wearing shoe frequently					
Yes	264(70.6)	123(46.6)	1	1	
No	110(29.4)	74(67.3)	2.49(1.56–3.98) **	0.67(0.34–1.33)	0.255
Abdominal pain					
Yes	198(52.9)	114(57.6)	1.52(1.01–2.29) **	1.54(0.89–2.67)	0.120
No	176(47.1)	83(47.2)	1	1	

**, statistically significant;

COR, crude odds ration; AOR, adjusted odds ratio; CI, confidence interval; STHs, soil-transmitted helminths

Microscopic examination of the stool specimens revealed that 67.9% (254/374) of the study participants were infected with one or more helminthic parasites. The most prevalent parasites among the school children was *A*. *lumbricoides* (44.4%), followed by *S*. *mansoni* (31%), *T*. *trichiura* (11%), and hookworm (7.7%). Other rare parasites detected were *Taenia* species (1.3%), *Hymenolepis* species (1.3%) and *E*. *vermicularis* (0.8%). The prevalence of infection with STHs was 52.4%.

The study participants harbored 1 to 4 different helminthic parasites, in which single infection occurred more frequently (42.2%). Multiple infections (polyparasitism) were detected in 25.7% of all 374 examined children and in 37.8% of those with any infection (254 children). The most frequent dual infections among the children was *A*. *lumbricoides* with *S*. *mansoni* (10.7%). The same rate of dual infections (3.5%) with *A*. *lumbricoides* and *T*. *trichiura* or hookworm were seen. The only observed triple infections (2.4%) was the combination of *A*. *lumbricoides*, *S*. *mansoni*, and *T*. *trichiura* (Table 2).

**Table 2:** Distribution of single and multiple helminthic infections among school children in southern Ethiopia, 2015.

Parasite	Frequency	Prevalence of infection (%)
**Single infection**	**158**	**42.2**
*A*. *lumbricoides* only	83	22.2
*S*. *mansoni* only	58	15.5
*T*. *trichiura* only	11	2.9
Hookworm only	6	1.6
**Double infection**	**84**	**22.5**
*T*. *trichiura* and *Taenia* species	1	0.3
*T*. *trichiura* and hookworm	2	0.5
*A*. *lumbricoides* and *Taenia* species	4	1.1
*A*. *lumbricoides* and *Hymenolepis* species	1	0.3
*A*. *lumbricoides* and *T*. *trichiura*	13	3.5
*A*. *lumbricoides* and hookworm	13	3.5
*A*. *lumbricoides* and *S*. *mansoni*	40	10.7
*S*. *mansoni* and *T*. *trichiura*	5	1.3
Hookworm and *Hymenolepis* species	4	1.1
*S*. *mansoni* and hookworm	1	0.3
**Triple infection**	**9**	**2.4**
*A*. *lumbricoides*, *T*. *trichiura* and *S*. *mansoni*	9	2.4
**Quadruple infection**	**3**	**0.8**
*A*. *lumbricoides*, *S*. *mansoni*, hookworm and *E*. *vermicularis*	3	0.8
**No helminthic infection**	**120**	**32.1**

Infection with STHs occurred in 60.4% of girls and in 52.7% of children aged between 11–15 years although difference by age was not found to be statistically significant. In bivariate analysis, gender, site of defecation at home, washing hands after return from latrine, washing hand before eating a meal, wearing shoe frequently, and a periodic trimming of fingers’ nail had significant association with geohelminths. In multivariable logistic regression analysis, children who defecated in an open-field were at higher risk of having soil-transmitted helminthiasis compared to those who used latrine (AOR, 3.6; 95% CI 1.6–8.0; p = 0.001). Moreover, children with no habit of washing their hand before eating a meal were five-fold more infected than those who always practiced hand washing (AOR, 5.0; 95%CI 2.15–11.7; p <0.001) (Table 1).

The distribution of *S*. *mansoni* infection with socio-demography and other risk factors is presented in Table 3. The school children were engaged in different activities related to the lake water including swimming (85%), washing clothes (86.9%), fishing (27.3%), and irrigational activities (39%). Boys (31.9%) had similar rate of *S*. *mansoni* infection compared to girls (29.5%). However, factors such as age, address, swimming in the lake, and engaging in irrigation-related activities significantly influenced the rate of infection. In multivariable logistic regression analysis, children aged 11–15 years (36.3%) had higher rate of *S*. *mansoni* infection compared to those aged 5–10 years (24.9%) (AOR, 1.97; 95% CI 1.22–3.19; p = 0.006). Moreover, the study participants from Finchawa (36.3%) were more predisposed to the infection than children from Tullo (AOR, 2.13; 95% CI 1.31–3.46; p = 0.002). Swimming in the lake (AOR, 2.73; 95%CI 1.20–6.17; p = 0.016) or involving in irrigation-related activities (AOR, 1.68; 95%CI 1.04–2.71; p = 0.034) put children at higher odds of contracting schistsomal infection.

**Table 3:** The distribution of schistosomiasis mansoni by socio-demography and other factors among school children in southern Ethiopia, 2015.

Characteristics	Total %	Number (%) *S*. *mansoni* infected	COR (95% CI)	AOR (95% CI)	P value
Sex					
Male	235(62.8)	75(31.9)	1.12(0.71–1.77)	-	-
Female	139(37.2)	41(29.5)	1		
Age (years)					
5–10	173(46.3)	43(24.9)	1	1	
11–15	201(53.7)	73(36.3)	1.72(1.10–2.70) **	1.97(1.22–3.19) **	0.006
Address					
Tullo	181(48.4)	46(25.4)	1	1	
Finch**a**wa	193(51.6)	70(36.3)	1.67(1.1–2.61) **	2.13(1.31–3.46) **	0.002
Fishing from lake					
Yes	102(27.3)	33(32.4)	1.1(0.67–1.77)	-	-
No	272(72.7)	83(30.5)	1		
Swimming in the lake					
Yes	318(85.0)	107(33.6)	2.65(1.25–5.61) **	2.725(1.20–6.17) **	0.016
No	56(15.0)	9(16.1)	1	1	
Washing clothes in the lake					
Yes	325(86.9)	105(32.3)	1.64(0.81–3.35)	0.98(0.44–2.16)	0.955
No	49(13.1)	11(22.4)	1	1	
Irrigational activities					
Yes	146(39.0)	56(38.4)	1.74(1.12–2.72) **	1.68(1.04–2.71) **	0.034
No	228(61.0)	60(26.3)	1	1	
Abdominal pain					
Yes	198(52.9)	66(33.3)	1.26(0.81–1.96)	-	-
No	176(52.4)	50(28.4)	1		

**, statistically significant;

COR, crude odds ration; AOR, adjusted odds ratio, CI, confidence interval

## Discussion

We assessed the current status of infections with *S*. *mansoni* and STHs among school children in a context where different preventive measures have been undertaken. The overall prevalence of any intestinal helminthic infection among the school children was 67.9%. This result showed still high rate of helminthiasis despite substantial reduction compared to the finding (91.5%) eight years back in the same study area [23]. The possible reasons for the observed reduction in rate of infection may be due to the intervention measures being undertaken through the health extension program. By engaging the community, health extension workers provide a community health care, which includes improving proper and safe excreta disposal systems, water supply safety measures, food hygiene and safety measures, healthy home environment, and personal hygiene in each *kebele*. Moreover, mass chemotherapy against STHs has been administered to under-five children since 2010, which might reduce the rate of infections in the school children, particularly among those who recently joined elementary schools as the risk of re-infection increases with time. The availability of well-constructed cement latrines and clean tap water supplies to the schools might also contribute to the observed difference. However, helminthiasis remained to be a major health concern among the school children and demands the intensification of intervention measures to adequately reduce the burden of the disease.

The species-wise distribution of geo-helminthic infections in the current study showed similar patterns where parasites such as *A*. *lumbricoides* (44.4%), *T*. *trichiura* (11%), and hookworms (7.7%) predominated in the study population. In comparison, the prevalence of trichuriasis (46.1%) and hookworm infection (29.7%) in our previous study were considerably higher [23]. However, there was no difference in rate of ascariasis (41.3%) between the two studies, which resulted in the prevalence of STHs (52.4%) in the current study to remain high like the previous STHs result (67.3%) [23]. Of course, factors including worm burden, fertility, and eggs’ resistance to adverse environmental conditions favor *A*. *lumbricoides* to cause heavy soil pollution and sustain the transmission cycle for a longer period of time. Thus, the implemented intervention measures that reduced the transmissions of other STHs were not found to be effective against *A*. *lumbricoides*. The current findings were in agreement with reports on ascariasis in southwest Ethiopia (Jimma) (39.5%) [21] and northwest Ethiopia (Gondar) (39.8%) [19], on trichuriasis in southern Ethiopia (Chencha) (9.7%) [31], and on hookworm infection in northwest Ethiopia (Lumame) (7.7%) [25] and Gondar (4.9%) [19]. However, contrasting higher rates of ascariasis (60.3%) [31], trichuriasis (47.6%) and hookworm infection (12.9%) [21], and lower rates of the respective infections (26.2% [25], 1.7% [19]), 2.2% [31]) were also shown in different localities in Ethiopia. Overall, the observed burden of infections with STHs among the school children may indicate heavy fecal contamination of the soil in the study area. The improper utilization of latrines at school and the practice of open-field defecation at home emphasize the importance of providing a required support to the children on how to properly utilize a latrine. STHs infection can also be reduced by educating and assisting the children to improve practices of washing hands after using a latrine and before a meal, and trimming their fingers’ nail contaminated with dirt.

The rate of infection with *S*. *mansoni* (31%) in the current study was significantly lower than the result in the previous study (70.4%), which encourages the need to sustain and strengthen intervention efforts to further impact its transmission dynamics. Among the children attending the two elementary schools, those in Finchawa were more infected than in Tullo, which perhaps be due to the proximity of the children’s dwelling to the lake side where irrigation-related activities were carried out. As also reported by others [26, 27], the higher infection rate among the children aged 11–15 years may be because they relatively had more contacts with the lake water while swimming or other activities. Children who had practices of swimming in the lake or involved in irrigation-related activities were shown to be having more infections compared to those who had no such practices. The significant association between schistosomal infection and swimming practice [22, 28, 32] or engaging in irrigational activities [26] was also reported by others.

The observed significant reduction in prevalence of schistosomiasis from the previous high rate to the current moderate rate was achieved in the absence of a deworming program. In contrast, despite the presence of deworming intervention targeted to under-five since 2010 in the study area, the transmission of ascariasis was not found to be impacted in the school children. However, evidences from different countries showed that both prevalence and intensity of STH infection and schistosomiasis fell over time with interventions that include deworming targeted to school children. For example, a report in Seychelles indicated delivering interventions, which comprised of deworming of school-age children, health education, and improvement in sanitation and water supply, reduced the number of STHs infected children by more than 87% and eliminated cases of heavy infection over a period of 5 years [33]. Moreover, administering mass chemotherapy to school children in Burundi resulted in reduction in prevalence of *S*. *mansoni* from 12.7% to 1.7% and prevalence of hookworm from 17.8% to 2.7% after 4 years [34]. Thus, introducing a mass deworming administration program against STHs and *S*. *mansoni* in school children in the study area may complement the existing interventions to further diminish the public health impact of helminthiasis.

In contexts where various helminthic infections are prevalent, the occurrence of multiple parasitic infections is a common phenomenon. Thus, a considerable proportion of children (25.7%) in the current study harbored multiple infections, although the rate was much lower than our previous report (59.7%). This reduction has significance in terms of reducing the nutritional and pathological effects that multiple infections might cause. The more frequent occurrence of dual infections with *A*. *lumbricoides* and *S*. *mansoni* may be due to the higher distribution of the two parasites among the children, which favors the mixing-up.

This study has some limitations to be considered while interpreting its results. First, a single stool sample was collected and analyzed, which may underestimate the prevalence of helminthiasis as the chance of detecting parasites increases with examining multiple samples. Second, parasites’ egg count, which indicates the intensity of infection, was not determined in the current study to comment on the impact it might have to sustain the transmission cycle and increase the severity of the disease. Third, the quality of data on risk factors of parasitic infection depends on the recalling ability of the children’s parents/guardians and their commitment to provide genuine information; thus, a recall and/or information bias might be introduced.

## Conclusion

The study showed a high prevalence of STHs and moderate rate of schistosomiasis. Thus, as recommended by the World Health Organization [2], mass deworming twice a year for STHs and once every two years for *S*. *mansoni* helps control the consequences of the infections to the level that they no longer constitute a public health burden. Moreover, as much of the exposure to helminthic infections was related to poor personal hygiene, unsanitary conditions and frequent contact with the lake water, those factors should be addressed adequately to sustain impact of a mass deworming administration program.

## Supporting information

S1 Questionnaire This is questionnaire in Sidamigna language. (DOCX)

S1 Data This is SPSS data of helminths. (SAV)
