# Supplementary material for: Infections with Schistosoma mansoni and geohelminths among school children dwelling along the shore of the Lake Hawassa, southern Ethiopia
Source: PLoS One. 2017 Jul 18;12(7):e0181547. doi: 10.1371/journal.pone.0181547 (PMC5515461; doi:10.1371/journal.pone.0181547)
Supplement: S1 Questionnaire — (DOCX) [file pone.0181547.s001.docx]

**Questionnaire (English Version)**

1. Name of the participant ----------------------- code number ---------------------------
2. Age -------
3. Sex ------
4. Is there a latrine at your home?
5. Yes B. no
6. Where does your child defecate at home?
7. Latrine B. open field
8. Does your child wash his/her hand after latrine using soap?
9. Yes B. no
10. Does your child wash his/her hand before meal using soap?
11. Yes B. no
12. Does your child wear shoe regularly?
13. Yes B. no
14. Does your child practice fishing in the lake?
15. Yes B. no
16. Does your child practice swimming in the lake?
17. Yes B. no
18. Does your child wash his/her clothes using lake water?
19. Yes B. no
20. Does your child practice irrigational activities by taking water from lake?
21. Yes B. no
22. Does your child feel abdominal discomfort?
23. Yes B. no
24. Does your child trim his/her finger periodically?
25. Yes B. no

**Questionnaire (Sidamigna Version)**

1. Xiin xaamote beeqqaanchi su’ma ___________________maaxooshshu kiiro________
2. Diro _____________________
3. Koo/Tee _______________________
4. Mene’ne shumate mini no? A. EE B. dino
5. Beeti’ne mama ofolanno? A. Shumate mine B. xawoho
6. Beeti’ne Shumate minni higanno woyiite angasi saamununni hayiishiranno?
7. EE B. dihayiishiranno
8. Beeti’ne Sagale itate albaanni angasi saamununni hayiishshinno?
9. EE B. dihayiishiranno
10. Beeti’ne Caamma duucha woyiite wodhano? A. EE B. diwodhono
11. Beeti’ne Garbunni qulxu’me amadano? A. EE B. dee’ni
12. Beeti’ne Garbu gildo daahano? A. EE B. dee’ni
13. Beeti’ne Uddanosi garbu wayiinni hayiishirano? A. EE B. dihayiishiranno
14. Beeti’ne Garbu wayiiini gorsu latishshi looso loosano? A. EE B. dee’ni
15. Beeti’ne Godowu xisso noosi? A. EE B. dinosi
16. Beeti’ne Culunqasi yanna yannate haransire mudhano? A. EE B. dee’ni
